# Crocetin inhibits Aβ aggregation in vitro and reduces ROS production and Aβ mediate cellular toxicity in neuronal SHSY5Y cells

**DOI:** 10.1002/alz.087283

**Published:** 2025-01-03

**Authors:** Pratap GK, Poornima Priyadarshini CG

**Affiliations:** ^1^ CSIR‐CFTRI, Mysore, Karnataka India

## Abstract

**Background:**

Alzheimer’s disease (AD) is a neurodegenerative disorder characterized by senile plaques, amyloid‐beta (Aβ), and neuroinflammation. The key targets in the treatment of AD are inhibiting the production of amyloid‐beta (Aβ), sphingomyelinase, and inflammation. Among the mechanisms, sphingolipids (specifically Ceramides) are recognized as distinctive mediators associated with the pathology of AD. Reportedly, alterations in sphingolipid metabolism and their metabolites result in mitochondrial dysfunction, autophagy impairment, dysregulation of amyloid‐beta, and disruption of neuronal homeostasis. This leads to increased production of reactive oxygen species (ROS), cytochrome c release, Bcl‐2 depletion, and caspase‐3 activation, primarily through modulation of intracellular signaling pathways, particularly those related to Akt/PKB kinase and mitogen‐activated protein kinases (MAPKs), exacerbating the progression of AD. Several natural compounds such as alkaloids, flavonoids, terpenoids, and phenolics have been reported to exhibit neuroprotective effects targeting various biomarkers including Aβ.

**Method:**

The present study investigates the inhibition of amyloid‐β aggregation, oxidative stress markers (ROS), CD spectra, sphingomyelinase and amyloidogenic activity in vitro of Crocetin, a carotenoid isolated from Gardenia gummifera fruit.

**Result:**

Our results show that crocetin was shown to inhibit aggregation of Aβ1‐40 up to>60%. Compared to Galantamine. Further, attenuation of Aβ1‐40‐induced neuronal toxicity in SH‐SY5Y cells by up to 50% was also observed along with the prevention of reactive oxygen species accumulation.

**Conclusion:**

Overall, these findings suggest that Crocetin may be a promising candidate for the management of AD

